# Comparative Evaluation of Oscillating Fiber Laser Welding and GMAW/MAG for SAE 1010 Agricultural Wheel Rims

**DOI:** 10.3390/ma19214746

**Published:** 2026-11-08

**Authors:** Willian Queiroz, Francisco Briones, Carola Martínez, William Haupt

**Affiliations:** 1 Postgraduate Program in Design and Manufacturing Processes (PPGPPF), Department of Mechanical Engineering, University of Passo Fundo (UPF), Br 285 km 292, Passo Fundo 99052-900, Brazil; 166705@upf.br (W.Q.); williamhaupt@upf.br (W.H.); 2 Escuela de Ingeniería Mecánica, Pontificia Universidad Católica de Valparaíso, Av. Los Carrera 01567, Quilpué 2430000, Chile; 3 Departamento de Ingeniería en Obras Civiles, Universidad de La Frontera, Temuco 4811230, Chile; carola.martinez@ufrontera.cl

**Keywords:** laser welding, GMAW/MAG welding, agricultural wheel rims, product development methodology, microstructure, Vickers microhardness, weld quality

## Abstract

This study evaluates oscillating fiber laser welding as an alternative to gas metal arc welding with an active shielding gas (GMAW/MAG) for manufacturing 4.25 mm thick SAE 1010 agricultural wheel rims. A systematic product development approach guided technology selection. The comparison included process energy, weld geometry, microstructure, grain size, Vickers microhardness, guided-bend testing, and industrial forming. Estimated GMAW/MAG heat input was 0.64 kJ/mm, whereas nominal incident laser energy was 0.019 kJ/mm; these quantities do not represent equivalent measurements of absorbed heat. Measurements from one transverse cross-section per welding condition showed a smaller weld-face width (5.70 vs. 12.97 mm), lower convexity (0.56 versus 3.93 mm), and a narrower heat-affected zone (0.85 versus 2.10 mm) for laser welding compared with GMAW/MAG. Less grain coarsening was observed in the laser-welded heat-affected zone. Mean fusion-zone hardness was 158.6 ± 18.9 HV1 for laser welding and 190.4 ± 11.0 HV1 for GMAW/MAG. Both conditions passed the guided-bend tests. All ten laser-welded specimens completed industrial forming, whereas two of ten GMAW/MAG specimens fractured during cone opening. The examined laser-welded cross-section exhibited a narrower weld profile than the examined GMAW/MAG cross-section. The laser-welding manufacturing route also eliminated the post-weld grinding operation under the investigated conditions.

## 1. Introduction

Agricultural wheel rims are structural components subjected to combinations of static, cyclic, and impact loads under demanding service conditions. Their manufacture generally involves cutting and longitudinally joining a low-carbon steel strip, followed by forming operations such as roll bending, expansion, profiling, and flanging. During these stages, the welded region undergoes substantial plastic deformation and must accommodate changes in curvature and cross-sectional geometry without cracking, joint opening, or unacceptable dimensional deviations. Metallurgical heterogeneity, unfavorable crystallographic texture, local thinning, oxide inclusions, and geometric discontinuities can promote strain localization and make the welded region a preferential failure site during forming [1,2,3,4]. Therefore, weld integrity must be evaluated not only in the as-welded condition but also throughout the subsequent manufacturing sequence.

Gas metal arc welding with an active shielding gas, commonly referred to as GMAW/MAG, is widely used to join structural and low-carbon steels because of its versatility, high deposition rate, accessibility, and suitability for automated production. However, its arc-based heat source distributes energy over a relatively large area and generally requires filler-metal addition. Depending on the process parameters and joint configuration, GMAW/MAG can produce a wide fusion zone, pronounced weld reinforcement, a wide heat-affected zone (HAZ), residual stresses, and geometric distortion [5,6,7]. The resulting thermal cycle may also generate heterogeneous microstructures and hardness gradients across the fusion zone, HAZ, and base material, potentially leading to nonuniform deformation during post-weld forming [6,7,8,9]. In addition, excessive weld reinforcement may require grinding before profiling, increasing processing time, material loss, and dimensional variability.

Laser beam welding (LBW) offers an alternative for applications requiring high productivity, dimensional accuracy, and localized thermal effects. The high-power density of a focused laser beam enables high travel speeds and deep penetration, producing welds with a high depth-to-width ratio and a narrow HAZ [7,10,11,12]. Compared with arc-based processes, LBW can substantially modify weld geometry, thermal history, solidification behavior, microstructure, and hardness distribution [7,10,13]. Nevertheless, the reduced nominal heat input associated with laser welding does not necessarily guarantee suitable joint performance. High cooling rates may promote localized hardening and the formation of non-equilibrium constituents, depending on the chemical composition, initial microstructure, peak temperature, and cooling conditions [8,9,14]. Consequently, laser-welded joints must be evaluated by considering weld geometry, microstructure, hardness distribution, weld imperfections, and manufacturing-induced deformation.

Diverse studies have examined laser welding of low-carbon steels by investigating the relationships among thermal conditions, microstructure, and mechanical properties. The high energy concentration of the laser enables narrow weld beads and a limited heat-affected zone (HAZ) [15]. However, rapid cooling may cause localized hardening and promote the formation of bainite or martensite, depending on the steel composition and welding conditions [16]. Therefore, these geometric advantages should be assessed alongside microstructural changes and their influence on joint properties.

Comparative studies of laser welding and GMAW in selected low-carbon and low-alloy steels have reported differences in HAZ width, weld geometry, and hardness distribution [16]. Under the investigated conditions, laser welding generally produced a more localized thermally affected region and, in some cases, reduced distortion [17]. Nevertheless, these advantages do not necessarily ensure better mechanical performance, as joint behavior also depends on chemical composition, sheet thickness, filler metal, joint configuration, and welding parameters [18].

These findings motivate an application-specific assessment of both technologies for SAE 1010 agricultural wheel rims, whose longitudinal welded joints must withstand subsequent forming operations. Although previous studies have investigated welding-related failures and forming behavior in wheel rims [3], the replacement of the existing industrial GMAW/MAG process with oscillating fiber laser welding requires validation under the relevant manufacturing conditions. The present study addresses this need by relating weld geometry, microstructure, and microhardness to guided-bend performance and subsequent industrial forming behavior.

The forming behavior of tailor-welded blanks has been extensively investigated for automotive applications. Previous studies have shown that weld geometry, mechanical property mismatch, HAZ softening or hardening, thickness differences, and anisotropy can affect strain localization, material flow, and fracture during forming [6,19,20]. However, these responses depend on the steel grade, sheet thickness, welding conditions, and loading path, limiting the direct transfer of conclusions between different products and material configurations [6,19]. Beam oscillation provides an additional means of controlling melt-pool geometry and thermal distribution by modifying fluid flow and the effective trajectory of the heat source. This approach may improve process stability, surface regularity, gap-bridging capability, and weld-pool homogeneity [21], which are relevant for industrial butt joints with variable fit-up conditions and dimensional tolerances.

Despite extensive research on laser welding of structural and automotive steels, its application to wheel-rim manufacturing remains comparatively underexplored. Previous investigations have primarily addressed flash or resistance butt welding, metallurgical defects, material banding, HAZ transformations, and cracking during the cold forming of automotive rims [1,2,3,4,22]. Limited information is available on replacing conventional GMAW/MAG with oscillating fiber laser welding in butt joints of cold-rolled SAE 1010 steel for agricultural wheel rims. In particular, an integrated comparison encompassing process energy, weld macrogeometry, HAZ characteristics, grain size, microhardness distribution, weld-quality criteria, guided-bend response, and validation through industrial forming operations remains insufficiently documented.

Therefore, this study evaluates the feasibility of replacing conventional GMAW/MAG with oscillating fiber laser welding to manufacture agricultural wheel rims from 4.25 mm thick SAE 1010 steel. The working hypothesis is that the laser process, with its higher energy concentration and travel speed, can reduce thermal and geometric discontinuities while maintaining the joint integrity required for severe post-weld forming. The two welding processes were compared in terms of process energy, penetration, weld geometry, HAZ and fusion-zone characteristics, grain size, Vickers microhardness, observable weld imperfections, and compliance with applicable quality criteria. Functional performance was subsequently evaluated through guided-bend testing and industrial roll bending, cone opening, and profiling operations.

## 2. Materials and Methods

### 2.1. Experimental Design

This study combined systematic product development principles with experimental validation in agricultural wheel-rim manufacturing. The initial stage identified failures in butt-welded joints during subsequent mechanical forming operations. Based on this diagnosis, technical requirements were defined for structural integrity, geometric stability, plastic deformation behavior, production repeatability, surface quality, and compliance with applicable standards.

The welding technology was selected using the methodology proposed by Pahl and Beitz [23], complemented by the project-planning principles described by Back et al. [24]. A weighted decision matrix was used to compare welding alternatives against the technical requirements identified for agricultural wheel-rim manufacturing. This analysis identified laser welding as a potential replacement for the conventional GMAW/MAG process, and its technical feasibility was subsequently evaluated experimentally.

The experimental program compared conventional gas metal arc welding with an active shielding gas (GMAW/MAG) and oscillating fiber laser beam welding (LBW) for manufacturing agricultural wheel rims. The comparison considered joint macrogeometry, heat-affected zone characteristics, microstructure, grain size, Vickers microhardness, weld imperfections, guided-bend response, and performance during subsequent industrial forming operations.

A total of 40 butt-welded joints were produced, comprising 20 GMAW/MAG joints and 20 laser-welded joints. After stabilization of the welding parameters, all joints were visually inspected for apparent welding discontinuities. Representative specimens were then selected from each production batch for subsequent analyses.

Quantitative weld-geometry characterization was based on one transverse cross-section from each welding condition. The resulting measurements were used for a descriptive comparison of the selected weld profiles, without statistical estimation of between-joint variability. Microstructural characterization and microhardness evaluation were performed on representative transverse cross-sections from each welding condition. Two independently welded specimens were evaluated for each welding process, with one transverse microhardness profile obtained from each specimen. Mechanical validation included three face-bend and three root-bend specimens for each welding process, whereas industrial forming validation was conducted using ten welded specimens per condition.

The experimental sequence comprised: (i) preparation and alignment of the steel sheets; (ii) production of GMAW/MAG and laser-welded butt joints; (iii) process-energy estimation; (iv) macrographic and geometric characterization; (v) optical microstructural analysis and grain-size measurement; (vi) Vickers microhardness profiling; (vii) visual inspection and weld-quality assessment; (viii) guided-bend testing; and (ix) validation through industrial roll bending, cone opening, and profiling.

### 2.2. Base Material

The base material was cold-rolled SAE 1010 low-carbon steel supplied by ArcelorMittal, Espírito Santo, Brazil, with a nominal thickness of 4.25 mm. This steel corresponds to the material currently used in the industrial manufacture of the agricultural wheel rims evaluated in this study.

Rectangular sheets measuring 400 mm × 500 mm × 4.25 mm were cut with their longitudinal direction perpendicular to the rolling direction. Before welding, the sheet edges were prepared by industrial cutting and mechanically cleaned to remove oil, oxides, and other surface contaminants. The sheets were assembled in a square butt-joint configuration without beveling and with a nominal root gap not exceeding 0.15 mm.

The chemical composition of the base material was determined by optical emission spectrometry using an Oxford Instruments spectrometer (Abingdon, UK). The measured chemical composition is presented in Table 1.

### 2.3. Production of Welded Joints

#### 2.3.1. GMAW/MAG Welding

The reference joints were produced using the GMAW/MAG process currently employed on the industrial manufacturing line. Welding was performed using a Panasonic Full Digital IGBT Pulse Controlled CO_2_ power source with a rated current of 500 A (Panasonic Connect Co., Ltd., Kadoma, Osaka, Japan).

The sheets were assembled in a square butt-joint configuration and rigidly clamped using a dedicated fixture to maintain alignment and minimize welding-induced distortion.

An ER70S-6 solid wire with a diameter of 1.6 mm was used as the filler metal. A shielding gas mixture of 90% Ar and 10% CO_2_ was supplied at a flow rate of 23 L/min. Welding was performed in a single pass using direct-current electrode-positive polarity.

The welding current and arc voltage were 285 A and 32 V, respectively, and the travel speed was 68 cm/min. The Arc Characteristic setting of the power source was set to 8, according to the industrial welding procedure adopted by the manufacturer. The contact-tip-to-work distance was maintained at 15 mm, corresponding to an electrode extension of approximately 12 mm. The torch was positioned perpendicular to the workpiece, with a 90° work angle and a 0° travel angle, and welding was performed in the flat position (PA).

The welding operation was fully automated to maintain constant travel speed and repeatable process conditions. Metal transfer occurred in spray mode under conventional constant-voltage operation. Pulsed current was not used, despite the pulse capability of the power source.

All parameters followed the industrial welding procedure specification currently adopted for agricultural wheel-rim production. The welding conditions remained constant throughout the experimental program to maintain consistent nominal settings and permit comparison with the laser welding process.

#### 2.3.2. Fiber Laser Welding

Laser welding was performed using a Han’s Laser HW1008 fiber laser welding system with a rated power of 3000 W and a nominal laser wavelength of 1080 ± 5 nm (Han’s Laser Technology Industry Group Co., Ltd., Shenzhen, Guangdong, China).

The laser beam was subjected to linear transverse beam oscillation. The programmed oscillation amplitude and frequency were 5.2 mm and 2000 Hz, respectively, as defined by the scanner controller. The focal position was set at 0 mm relative to the upper surface of the sheet. Based on the optical configuration of the welding head, the focused laser spot diameter was approximately 0.3 mm. The optical system used a lens with a focal length of 150 mm, resulting in a working distance of approximately 150 mm.

**Figure 1:**
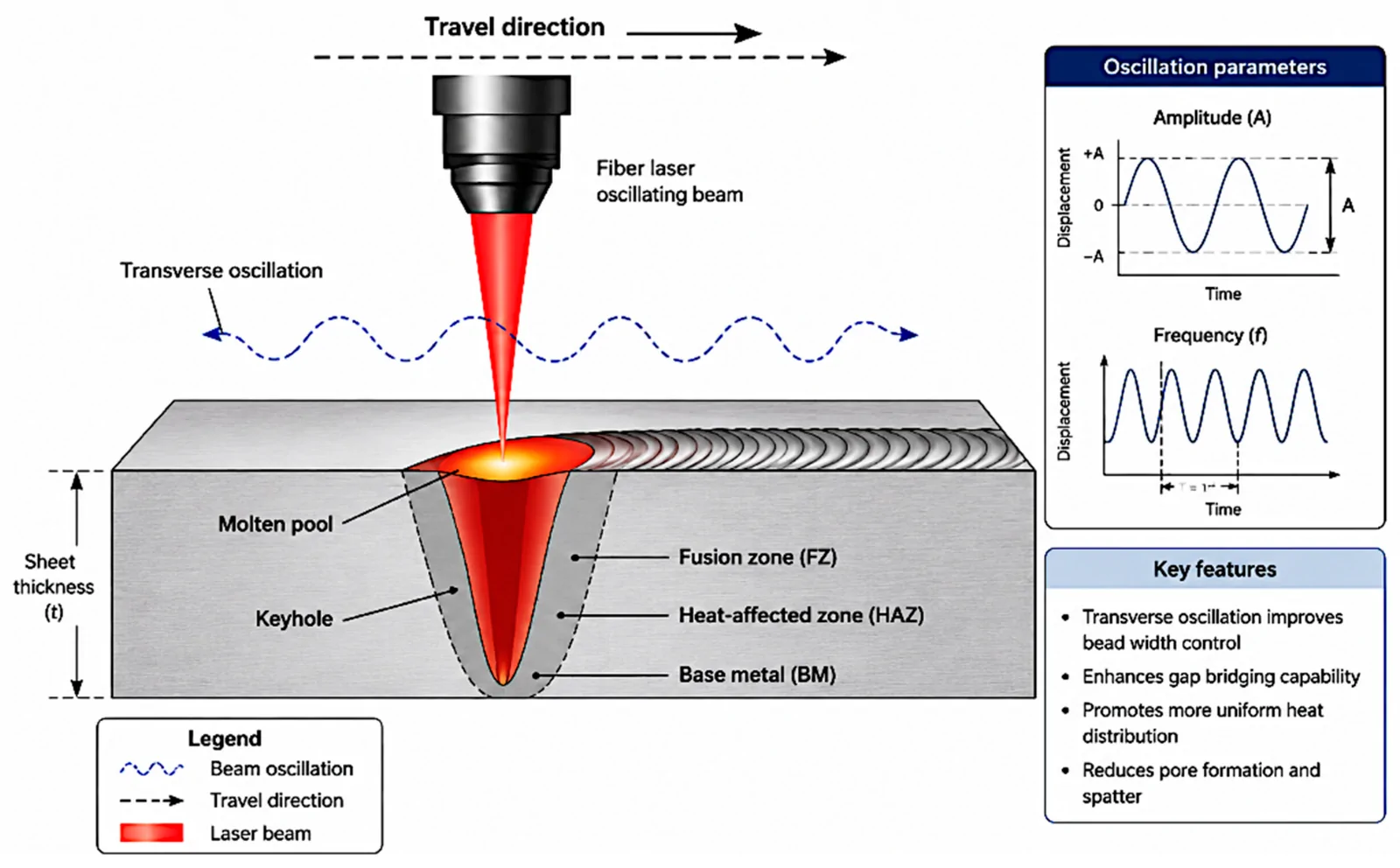
Schematic representation of the oscillating fiber laser welding process adopted in this study.

Figure 1 schematically illustrates the oscillating fiber laser welding process, including the welding direction, transverse beam oscillation, and the principal geometric parameters associated with beam movement.

High-purity nitrogen (99.999%) was used as the shielding gas at a flow rate of 20 L/min. The gas was supplied through a coaxial nozzle with an internal diameter of 12 mm to protect the molten pool and minimize atmospheric contamination.

The sheets were assembled in a square butt-joint configuration with a controlled root gap of less than 0.15 mm. The welding direction was perpendicular to the rolling direction of the steel sheet. A dedicated mechanical fixture with locking clamps was used to maintain alignment and minimize angular distortion during welding. Copper backing plates were positioned at the beginning and end of the weld path to stabilize laser-beam entry and exit and reduce the occurrence of start- and end-crater defects.

The welding operation was performed using a six-axis industrial robotic arm (São Paulo, Brazil) at a programmed travel speed of 155 mm/s to control welding-head positioning and travel speed throughout the experimental program.

**Figure 2:**
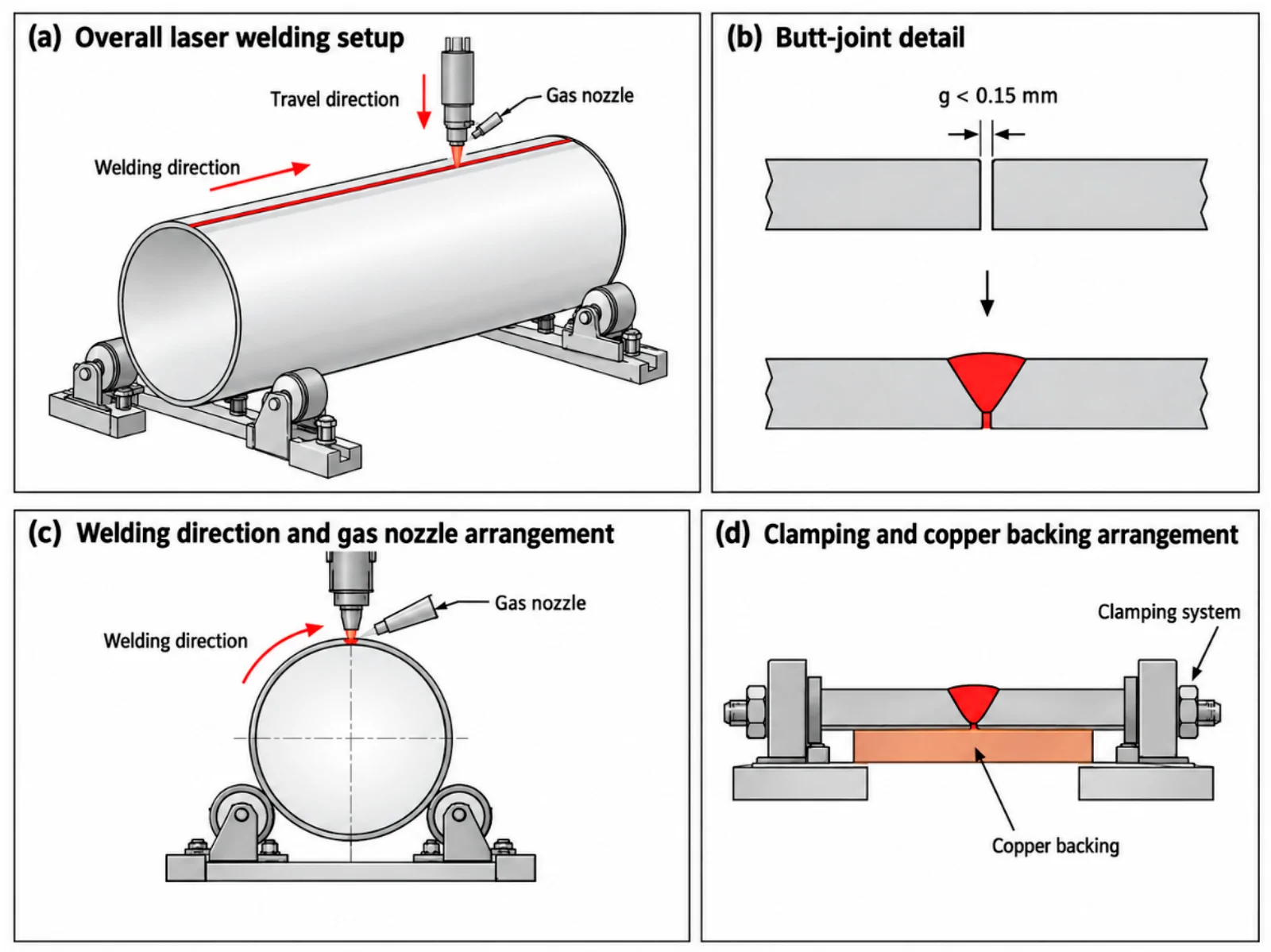
Schematic representation of the experimental laser welding configuration used for the preformed SAE 1010 agricultural wheel rim: (**a**) overall welding setup; (**b**) square butt-joint geometry with a controlled root gap of less than 0.15 mm; (**c**) welding direction and shielding-gas nozzle arrangement; and (**d**) mechanical clamping system and copper backing used to maintain joint alignment and stabilize the welding process.

The laser welding parameters were established through preliminary exploratory welding trials, conducted to identify suitable operating conditions for joining the SAE 1010 steel sheets. The selected parameters were subsequently maintained throughout the experimental program to ensure consistent nominal welding conditions (Figure 2).

### 2.4. Welding Parameters and Heat-Input Estimation

The principal parameters employed for both welding processes are summarized in Table 2.

The nominal heat input per unit length for the GMAW/MAG process was estimated according to ISO/TR 17671-1:2002 [25] using Equation (1):

(1) $$Q_{\frac{\mathrm{GMAW}}{\mathrm{MAG}}}=\frac{\eta \;\times \;V\;\times \;I\;}{v}10^{-3}\;$$

where $Q_{\frac{\mathrm{GMAW}}{\mathrm{MAG}}}$ is the estimated heat input per unit length (kJ/mm), V is the arc voltage (V), I is the welding current (A), v is the welding speed (mm/s), and η is the thermal efficiency factor. A value of 0.80 was adopted for GMAW/MAG in accordance with ISO/TR 17671-1:2002 [25]. The factor 10^−3^ converts J/mm to kJ/mm.

For laser beam welding, the nominal incident laser energy per unit length was calculated using Equation (2):

(2) $$E_{\mathrm{LBW}}=\frac{n\;\times \;P}{v}\;10^{-3}$$

where *E_LBW_* is the nominal incident laser energy per unit length (kJ/mm), *P* is the laser power (W), and *v* is the welding speed (mm/s). The factor 10^−3^ converts J/mm to kJ/mm.

Because laser absorptance depends on material properties, welding speed, beam characteristics, and keyhole behavior, no fixed thermal-efficiency factor was applied to the laser-welding calculation. Previous calorimetric investigations of high-power fiber laser welding have demonstrated that laser absorptance can vary substantially with processing conditions [26]. Therefore, the laser value reported here represents the incident energy supplied per unit weld length rather than the effective absorbed heat input.

Using the parameters adopted in this study, the estimated GMAW/MAG heat input was 0.64 kJ/mm, whereas the nominal incident laser energy was approximately 0.019 kJ/mm.

### 2.5. Specimen Extraction and Metallographic Preparation

Transverse specimens were extracted from the central region of each welded joint using a water-cooled metallographic cutting machine (São Paulo, Brazil) to minimize heat generation during sectioning. The weld start and end regions were excluded to avoid transient welding effects. The extracted cross-sections were used for macrographic, microstructural, and weld-geometry characterization.

Metallographic preparation was performed directly on the transverse cross-sections according to ASTM E3, while chemical etching followed ASTM E407. The specimens were ground using silicon carbide abrasive papers with grit sizes of 180, 320, 600, 800, and 1200. They were subsequently polished using 3 μm and 1 μm diamond suspensions until a mirror-like surface finish was obtained.

The polished surfaces were etched with a 2 vol.% Nital solution for approximately 10 s, rinsed with ethanol, and dried with warm air.

Macrographs were acquired using a Zeiss Stemi 2000-C stereomicroscope equipped with Zeiss ZEN image-acquisition software (2016). Optical micrographs were obtained using an Olympus BX51M microscope (Olympus Corporation, Tokyo, Japon) to examine the base metal (BM), heat-affected zone (HAZ), fusion boundary (FB), and fusion zone (FZ) produced by both welding processes. The images were used for qualitative microstructural characterization and quantitative grain-size measurements according to ASTM E112 [27].

### 2.6. Macrographic Examination and Weld Geometry Characterization

One transverse cross-section from each welding condition was selected for quantitative geometric characterization. Following metallographic preparation and chemical etching, the cross-sections were imaged under calibrated optical magnification.

Weld penetration, weld-face width, bead convexity, and HAZ width were measured using ImageJ software (2018) (National Institutes of Health, Bethesda, MD, USA). The measurements reported were obtained from these individual cross-sections (n = 1 per welding condition). Consequently, the results are presented as descriptive measurements without standard deviations or statistical comparisons between welding processes.

Full penetration was defined as continuous fusion through the local sheet thickness. The reported penetration depths were measured from the analyzed transverse macrographs; however, their interpretation relative to the nominal sheet thickness requires consideration of the local thickness, the measurement endpoints, and the image calibration. The HAZ boundary was identified as the metallographically visible transition between the thermally altered region and the unaffected base metal. The HAZ width was measured on both sides of the weld, and the arithmetic mean of these measurements was used to represent each individual cross-section.

### 2.7. Microstructural Characterization and Grain-Size Measurement

The microstructures of the base metal (BM), heat-affected zone (HAZ), and fusion zone (FZ) were evaluated by optical microscopy. The analysis focused on the ferrite–pearlite microstructure of the BM, grain refinement and coarsening across the HAZ, and the solidification morphology of the FZ produced by each welding process.

Microstructural observations were performed on one representative transverse cross-section from each welding condition, selected based on the macrographic analysis.

Grain-size measurements were conducted according to ASTM E112 [27] using the mean linear intercept method. Five non-overlapping fields were analyzed in each selected region using calibrated optical micrographs.

Because the FZ exhibited a directional solidification morphology, ASTM E112 [27] grain-size values were not assigned to this region, as reliable identification of equiaxed grains was not possible. Therefore, grain-size comparisons were restricted to the BM and the clearly defined HAZ.

### 2.8. Vickers Microhardness Testing

Vickers microhardness measurements were performed according to ASTM E384 [28] using a Shimadzu HMV-G20ST microhardness tester (Shimadzu Corporation, Kyoto, Japan). A test load of 1 kgf was applied for 15 s, corresponding to the HV1 test condition.

Two independently welded specimens were evaluated for each welding process, with one transverse microhardness profile obtained from each specimen. Each profile consisted of successive indentations spaced 0.5 mm apart, extending across the base metal, heat-affected zone, and fusion zone. The measurements were used to characterize the microhardness distribution in the different metallurgical regions of the welded joints.

**Figure 3:**
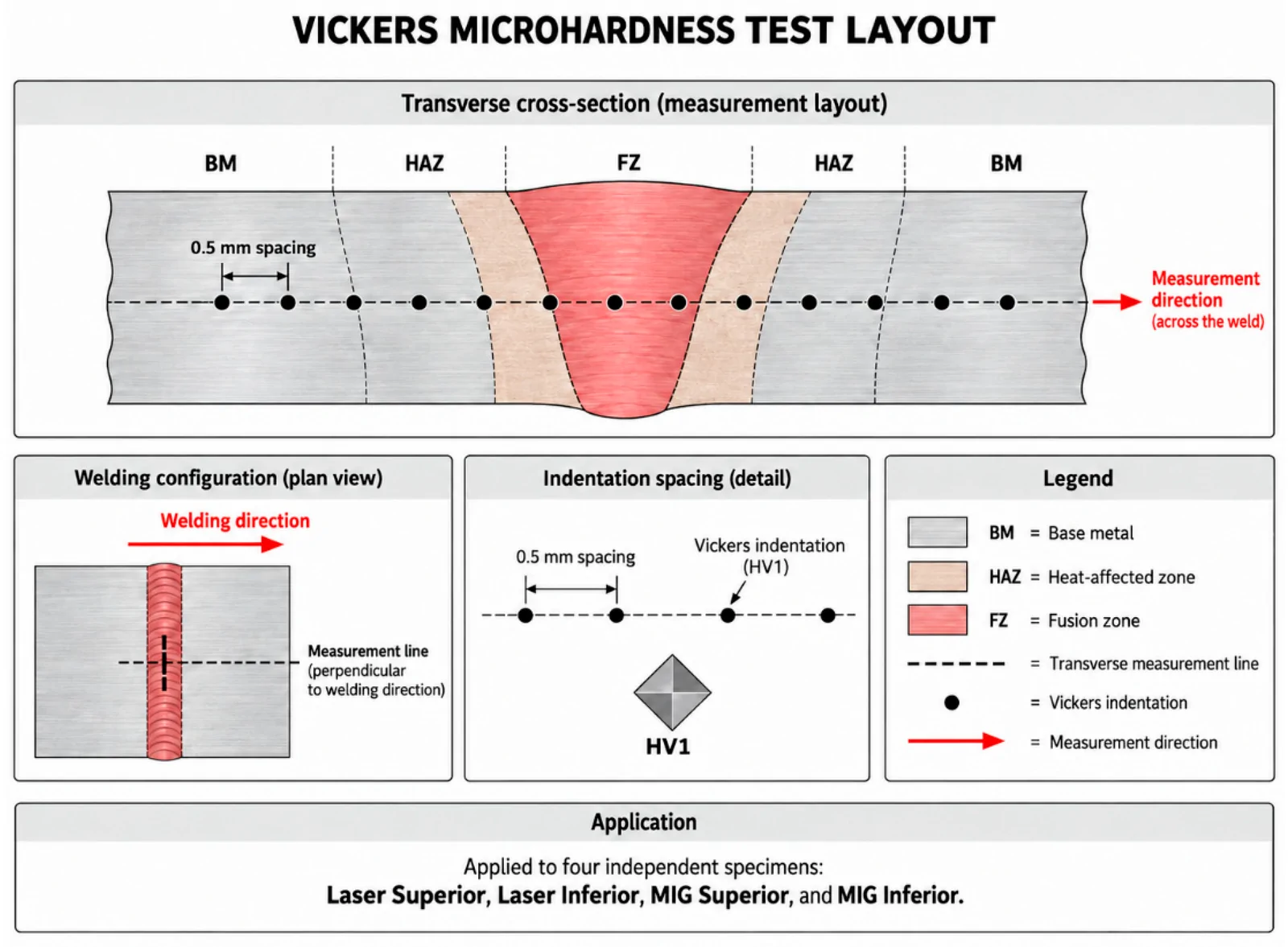
Schematic representation of the Vickers microhardness measurement procedure. One transverse HV1 microhardness profile was obtained from each of two independently welded specimens per welding process. Each profile extended across the BM–HAZ–FZ–HAZ–BM regions, with consecutive indentations spaced 0.5 mm apart.

One transverse microhardness profile was obtained from each specimen on a polished cross-section oriented perpendicular to the welding direction. The measurement line was positioned approximately at the mid-thickness of the welded joint, as schematically illustrated in Figure 3.

The indentations were distributed continuously across the welded joint, extending from the base metal (BM) on one side, through the heat-affected zone (HAZ) and fusion zone (FZ), to the HAZ and BM on the opposite side. A center-to-center spacing of 0.5 mm was maintained between consecutive indentations throughout each profile.

The metallurgical regions and HAZ boundaries were identified by optical examination of the transverse cross-sections after etching with 2 vol.% Nital. This procedure was used to correlate the indentation positions with the BM, HAZ, and FZ regions and to ensure consistent interpretation of the microhardness profiles for both welding processes.

### 2.9. Guided-Bend Testing

Guided-bend tests were performed on transverse face-bend and root-bend specimens extracted from GMAW/MAG- and laser-welded joints. The specimen geometry followed ASME Section IX, QW-462.3(a) [29], taking into account the dimensions of the welded sheets. Three face-bend and three root-bend specimens were tested for each welding process according to ASTM E190 and the requirements of ASME Section IX [29].

Before testing, the weld reinforcement was removed by grinding to obtain a surface flush with the base material. The tests were conducted using a Schenck UPM 200 universal testing machine with calibration certificate No. 10182/23. A cylindrical former with a diameter of 19 mm and a support spacing of 32 mm was used.

The specimens were bent through an angle of 180°, with the weld centered in the deformation region. Testing was performed under laboratory conditions at approximately 20 °C.

After bending, the convex surface of each specimen was visually examined for open discontinuities according to ASME Section IX [29], clauses QW-162 and QW-163. Specimens were considered acceptable when no open discontinuity exceeding 3 mm, or 1/8 in., was observed.

### 2.10. Validation by Industrial Forming

The functional performance of the welded joints was evaluated by reproducing the forming sequence used in the industrial manufacture of agricultural wheel rims. Ten welded specimens from each welding process were subjected to the complete industrial forming sequence.

**Figure 4:**
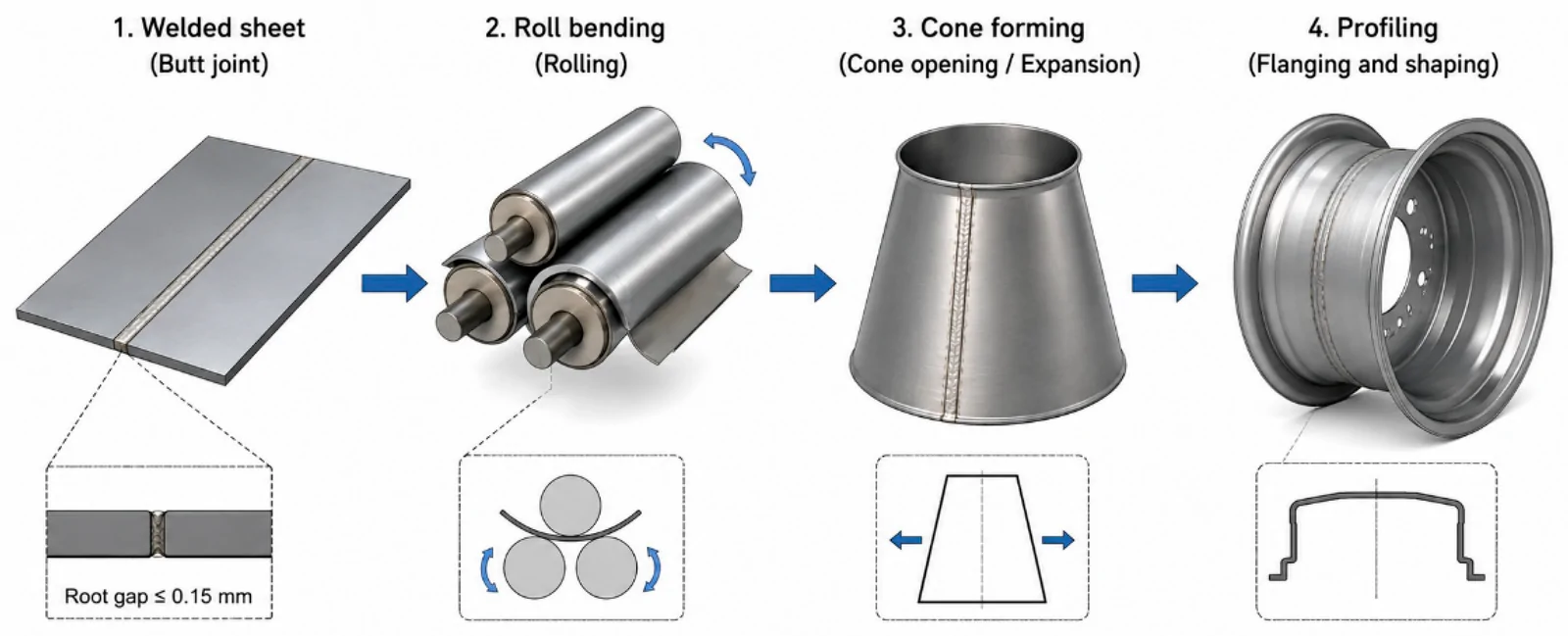
Schematic illustration of the manufacturing sequence used for the industrial validation of welded agricultural wheel rims.

The specimens were sequentially subjected to roll bending, cone opening, and profiling under the same operating conditions used on the industrial production line, as illustrated in Figure 4. The forming parameters, including machine settings, roll displacement, forming speed, and dimensional tolerances, were defined according to the manufacturer’s standardized industrial procedure. Owing to industrial confidentiality, the specific numerical values of these parameters are not disclosed.

After each forming stage, the welded region was visually inspected and dimensionally evaluated for cracking, joint opening, geometric instability, exposed porosity, and other discontinuities associated with plastic deformation.

The acceptance criteria were based on the absence of visible defects that could compromise the continuity of the welded joint or interfere with subsequent manufacturing stages. These defects included cracking, joint opening, excessive geometric distortion, exposed porosity, and other forming-related discontinuities.

The experimental procedures and corresponding standards or reference methods are summarized in Table 3.

## 3. Results and Discussion

### 3.1. Welding-Process Selection and Experimental Validation Strategy

The welding-process alternatives were initially compared using the systematic product development approach adopted in this study. The industrial problem involved failures and dimensional instability in butt-welded regions during subsequent forming of agricultural wheel rims. Therefore, the alternatives were evaluated not only by their as-welded characteristics but also by the functional requirements imposed by roll bending, cone opening, profiling, and flanging.

The decision matrix included six essential criteria: heat-affected zone reduction, penetration control, integrity after forming, process repeatability, hardness variation, and ease of industrial integration. Each criterion was assigned a weight according to its relevance to the manufacturing process, and each welding alternative was rated on a scale from 1 to 5. The weighted score for each criterion was calculated by multiplying its weight by the corresponding rating.

The criterion weights and alternative ratings were assigned by the author as an application-specific engineering assessment. HAZ reduction, penetration control, and integrity after forming received a weight of 5 because of their relevance to joint integrity during manufacturing. Repeatability and hardness variation received a weight of 4 because they influence production consistency and local deformation behavior. Ease of industrial integration received a weight of 3, reflecting the priority given to joint performance over implementation considerations. The alternatives were rated from 1 (very poor) to 5 (very good) according to their expected suitability for each criterion.

The maximum ratings assigned to laser welding reflected anticipated advantages for the proposed manufacturing route. Localized heating and beam control were expected to limit HAZ extension and facilitate penetration control, while reduced thermal exposure was considered potentially favorable for limiting hardness variation and maintaining integrity during forming. Controlled beam positioning and travel speed supported the expected repeatability rating. The industrial-integration rating reflected the potential for robotic automation of the proposed welding operation, rather than an experimentally established integration advantage over the existing automated GMAW/MAG process.

As shown in Table 4, these assumptions resulted in the highest weighted score for laser welding. The ratings represent engineering expectations, not measured performance or proof of superiority in every criterion. The matrix therefore served to prioritize a technology for investigation, while manufacturing feasibility was assessed separately through weld characterization, guided-bend testing, and industrial forming.

The optimized GMAW/MAG alternative represented a conceptual improvement of the existing process through adjustments to welding parameters and joint control, aimed at reducing excessive weld reinforcement, limiting HAZ extension, and improving consistency during subsequent forming. Its intermediate ratings reflected the anticipated benefits of these adjustments while retaining the same arc-based welding technology. These ratings were engineering estimates; no optimized parameter set was implemented or experimentally validated in this study.

### 3.2. Heat Input and Process-Energy Comparison

Table 5 summarizes the estimated energy inputs for the two welding processes. The estimated GMAW/MAG heat input was 0.64 kJ/mm, whereas the nominal incident laser energy per unit length was 0.019 kJ/mm. The estimated GMAW/MAG heat input was approximately 34 times the nominal incident laser energy per unit length. This comparison should be interpreted with caution, since the GMAW/MAG value includes a thermal efficiency factor of 0.80, whereas the laser value does not include an absorptance correction.

For the GMAW/MAG process, the energy input per unit length was calculated considering a thermal efficiency factor of 0.80. For laser welding, the reported value corresponds to the nominal incident laser energy per unit length, without applying a fixed thermal efficiency factor.

The difference resulted primarily from the welding speeds and the characteristics of the heat sources. GMAW/MAG welding combined arc heating with filler-metal deposition at a travel speed of 11.33 mm/s, whereas laser welding employed a concentrated heat source, transverse beam oscillation, and a substantially higher travel speed of 155 mm/s.

These values should be interpreted as comparative energy estimates rather than direct measurements of the energy absorbed by the material. The GMAW/MAG calculation included the adopted thermal efficiency factor, whereas the laser calculation used the nominal incident laser power and welding speed. For laser welding, P/v expresses incident energy per unit joint length based on the forward travel speed. Beam oscillation redistributes this energy spatially, but it does not require replacing the forward travel speed with the instantaneous scanning speed in this definition. Variations in laser absorptance were not quantified. These energy estimates provide context for discussing the observed geometric and microstructural differences, while recognizing that they do not quantify the relative heat absorbed by the material.

### 3.3. Weld Macrogeometry and Dimensional Stability

**Figure 5:**
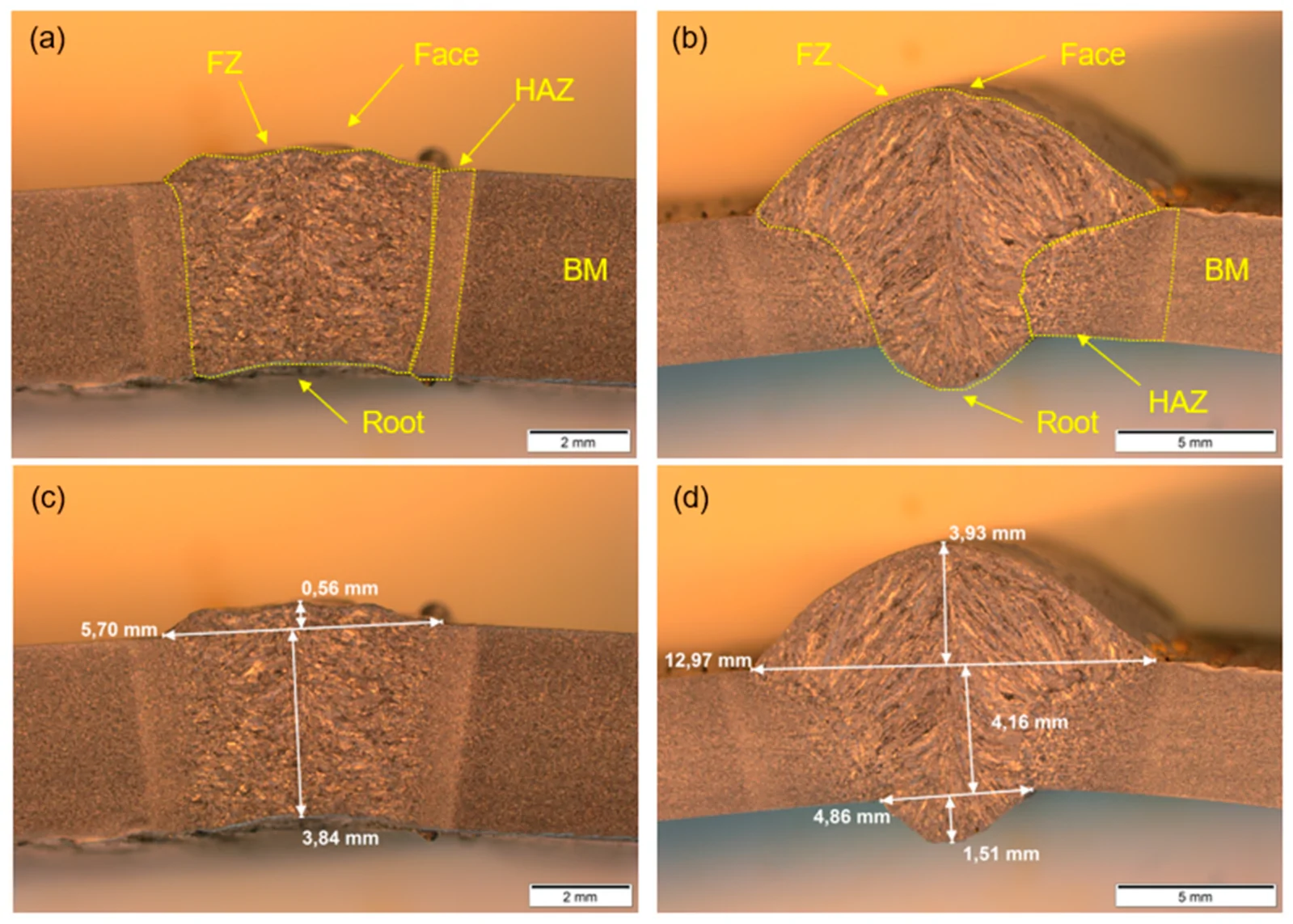
Transverse macrographs and geometric measurements of the analyzed welded joints: (**a**) laser-welded cross-section with metallurgical regions identified; (**b**) GMAW/MAG-welded cross-section with metallurgical regions identified; (**c**) dimensional measurements of the laser-welded cross-section; and (**d**) dimensional measurements of the GMAW/MAG-welded cross-section. BM: base metal; HAZ: heat-affected zone; FZ: fusion zone.

The transverse macrographs selected for quantitative analysis showed distinct weld geometries for the GMAW/MAG and laser-welded conditions. Figure 5 presents the analyzed cross-sections, and their corresponding geometric measurements are reported in Table 6. Because one cross-section was quantitatively evaluated per welding condition, these results provide a descriptive comparison rather than an assessment of between-joint variability.

The measured penetration depths were 3.84 mm for laser welding and 4.16 mm for GMAW/MAG, compared with a nominal sheet thickness of 4.25 mm. These values correspond to approximately 90.4% and 97.9% of the nominal thickness, respectively. However, the local sheet thickness at the analyzed cross-sections and the original measurement calibration require verification. Therefore, the recorded penetration depths alone cannot establish whether complete fusion through the local sheet thickness was achieved. The laser-welded cross-section exhibited a weld-face width of 5.70 mm, whereas the GMAW/MAG cross-section showed a width of 12.97 mm, corresponding to an approximately 56% smaller value in the analyzed laser-welded section.

The measured HAZ width was 0.85 mm for the laser-welded cross-section and 2.10 mm for the GMAW/MAG cross-section, representing an approximately 59.5% difference between the analyzed sections. This observation is consistent with the more localized thermal interaction expected under the investigated laser-welding conditions. However, the measurements do not establish the variability of HAZ width across independently welded joints.

Weld convexity was 0.56 mm in the laser-welded cross-section and 3.93 mm in the GMAW/MAG cross-section, corresponding to an approximately 85.7% smaller value in the analyzed laser-welded section. A distinct reinforcement dimension could not be reliably measured for the laser-welded cross-section, whereas a reinforcement of 1.51 mm was measured for GMAW/MAG. The lower convexity observed in the laser-welded section is relevant to the evaluated manufacturing route, in which excessive weld reinforcement may require grinding before subsequent forming operations.

No visible surface discontinuities were identified in the analyzed cross-sections. Overall, the selected laser-welded cross-section exhibited a narrower weld profile, lower convexity, and a more restricted HAZ than the selected GMAW/MAG cross-section. These geometric observations should be interpreted together with the independent guided-bend and industrial-forming results rather than as statistical evidence of geometric repeatability.

### 3.4. Microstructural Evaluation and Grain-Size Response

**Figure 6:**
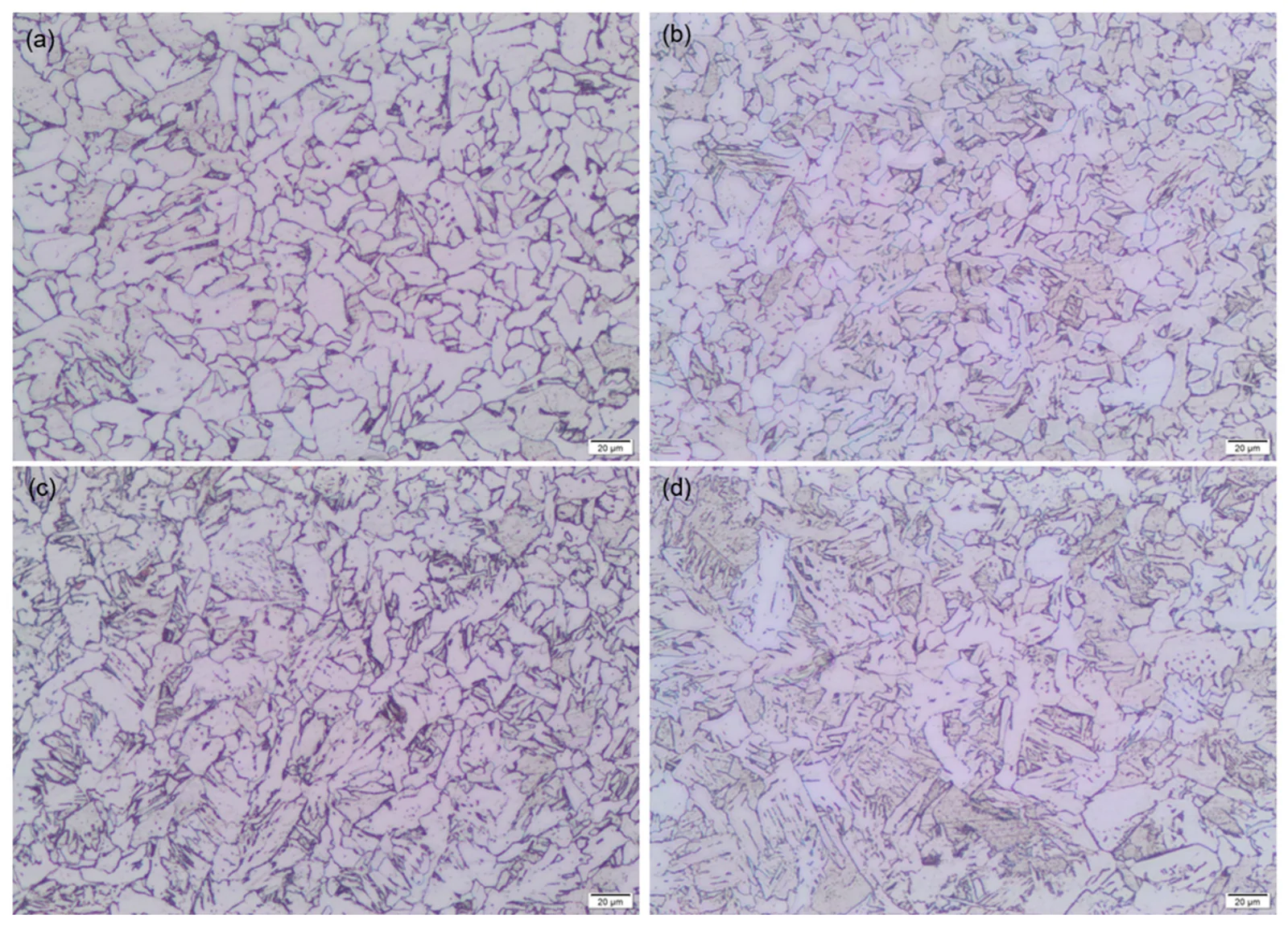
Optical micrographs of the base metal and heat-affected zone: (**a**) laser welding, BM; (**b**) laser welding, HAZ; (**c**) GMAW/MAG, BM; and (**d**) GMAW/MAG, HAZ. All micrographs were acquired at the same magnification. BM: base metal; HAZ: heat-affected zone.

Optical micrographs revealed the characteristic ferrite–pearlite microstructure of the SAE 1010 base metal. Representative micrographs of the base metal (BM) and the heat-affected zone (HAZ) are shown in Figure 6, with images 6a and 6b referring to laser welding and images 6c and 6d referring to GMAW welding. The corresponding grain-size measurements, obtained according to ASTM E112 [27], are summarized in Table 7. The reported standard deviations describe local variability among the five fields examined within each selected region, rather than variability between independently welded joints.

The laser-welded joint exhibited only minor differences in grain size between the base metal and the HAZ. The mean intercept length was 8.02 ± 0.30 µm in the base metal and 7.59 ± 0.35 µm in the HAZ, while the corresponding ASTM grain numbers were 10.64 ± 0.10 and 10.80 ± 0.12, respectively. Given the magnitude and uncertainty of these measurements, no appreciable grain coarsening was detected in the analyzed laser-welded HAZ.

In contrast, the GMAW/MAG joint exhibited pronounced grain coarsening in the HAZ. The mean intercept length increased from 8.25 ± 0.40 µm in the base metal to 16.40 ± 1.80 µm in the HAZ, corresponding to an increase of approximately 99%. Consequently, the ASTM grain number decreased from 10.55 ± 0.11 to 8.80 ± 0.25. These results indicate that the GMAW/MAG thermal cycle produced more extensive microstructural modification in the region adjacent to the fusion zone.

Grain-size measurements were restricted to the base metal and HAZ because the fusion zone exhibited a directional solidification morphology for which an ASTM E112 [27] grain-size number could not be reliably assigned. The fusion zones were therefore evaluated qualitatively by optical microscopy. Laser welding was performed autogenously, whereas GMAW/MAG employed ER70S-6 filler wire. Thus, the GMAW/MAG fusion zone resulted from dilution between the base and filler metals, while the laser fusion zone solidified from the molten base material. However, no compositional or phase analyses were performed to quantify the influence of filler-metal dilution on the observed microstructures.

Overall, the grain-size results were consistent with the macrographic measurements presented in Section 3.3. The localized thermal cycle associated with laser welding restricted measurable microstructural modification in the HAZ, whereas the broader thermal field generated by GMAW/MAG promoted more pronounced grain coarsening. Similar trends have been reported for laser welding of low-carbon steels, although the magnitude of the microstructural changes depends on the material, welding parameters, sheet thickness, and cooling conditions [7,9,10].

### 3.5. Vickers Microhardness Profile

**Figure 7:**
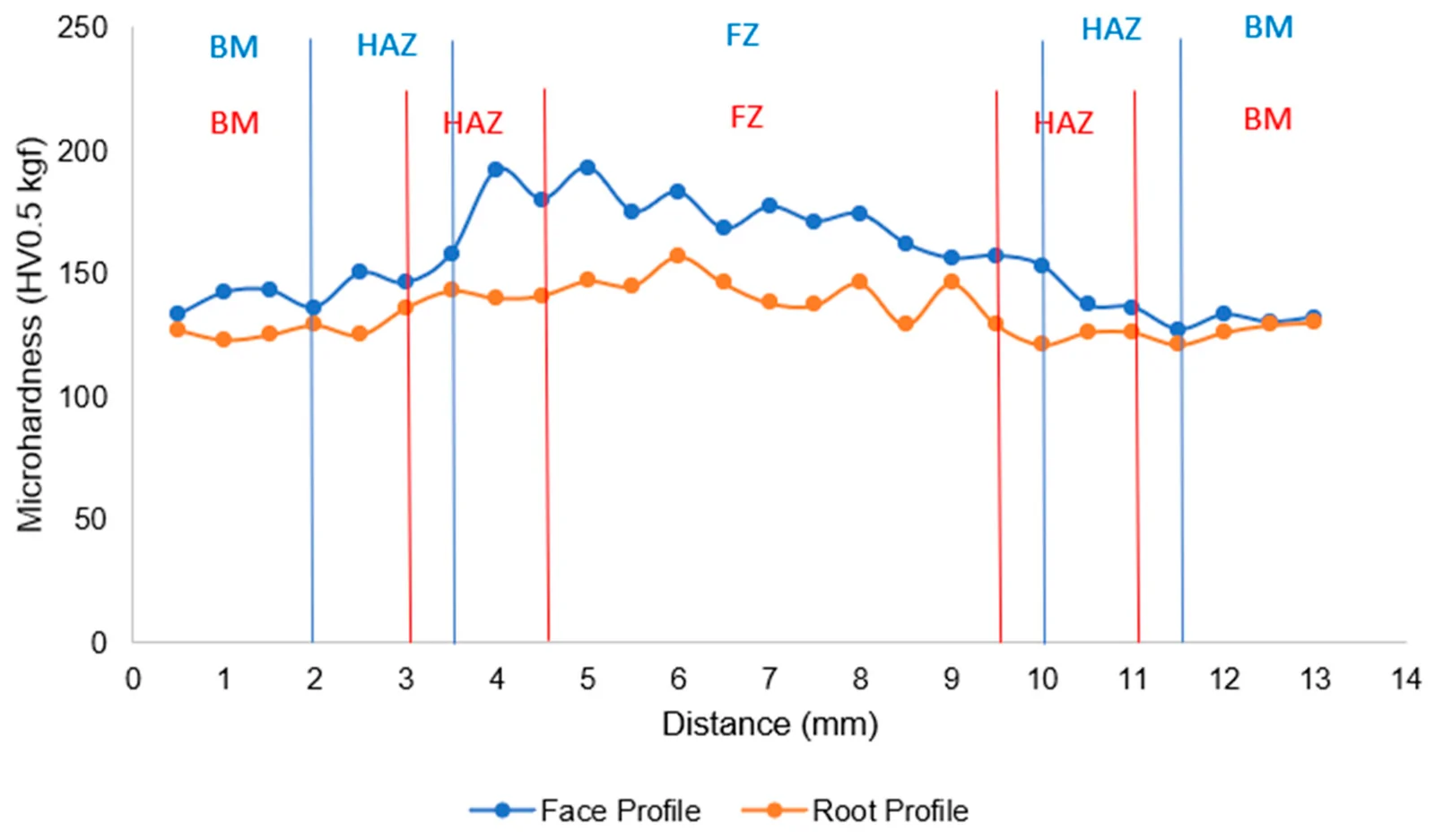
Transverse HV1 microhardness profiles obtained from two independent laser-welded specimens. The profiles extend across the base metal (BM), heat-affected zone (HAZ), fusion zone (FZ), HAZ, and base metal on the opposite side of the joint, with consecutive indentations spaced 0.5 mm apart.

**Figure 8:**
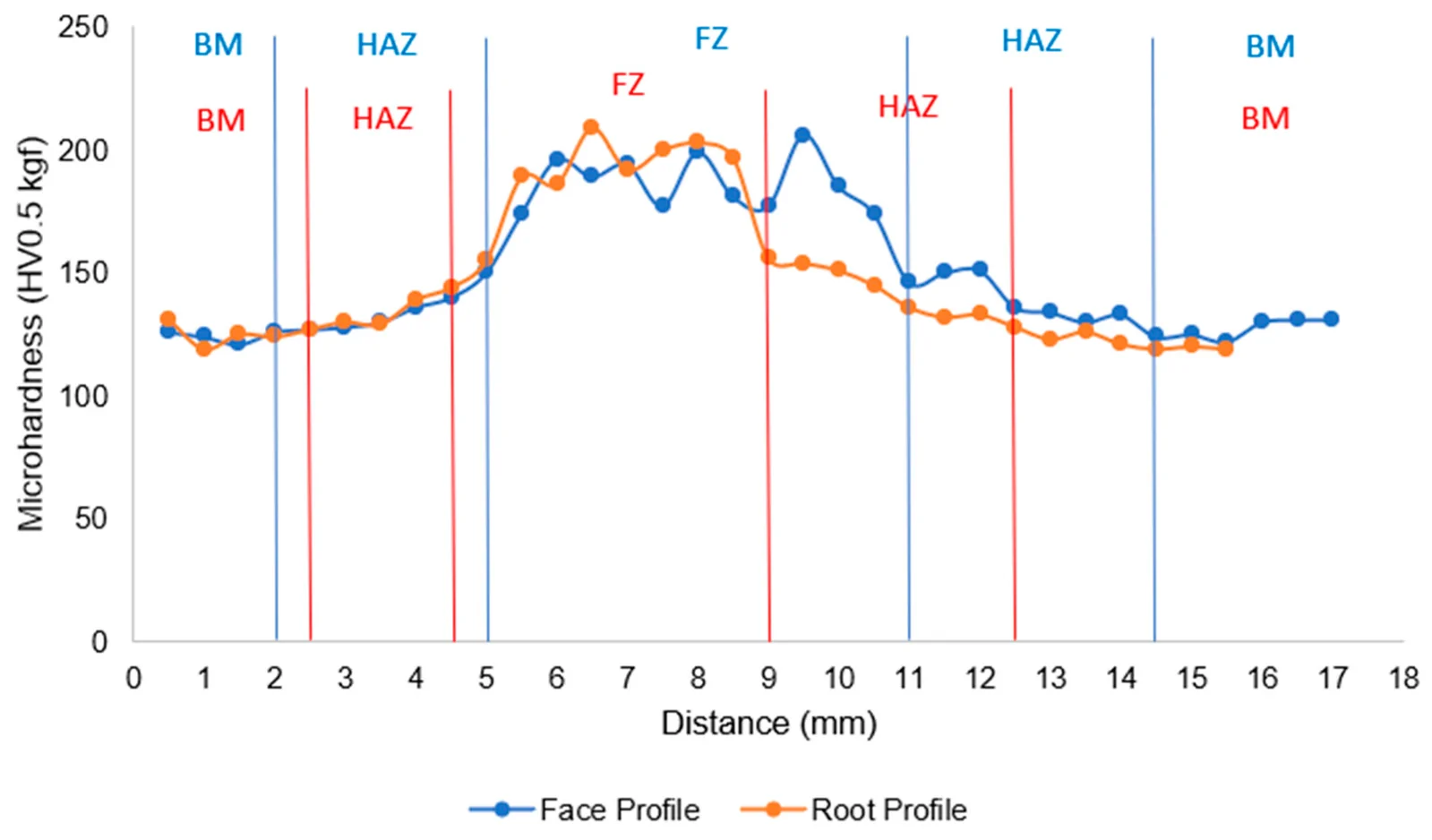
Transverse HV1 microhardness profiles obtained from two independent GMAW/MAG-welded specimens. The profiles extend across the base metal (BM), heat-affected zone (HAZ), fusion zone (FZ), HAZ, and base metal on the opposite side of the joint, with consecutive indentations spaced 0.5 mm apart.

Table 8 summarizes the Vickers microhardness values measured across the welded joints, and Figure 7 and Figure 8 present the corresponding transverse profiles.

The mean base-metal hardness values were relatively similar between the two welding conditions. For laser welding, the mean hardness values were 131.9 ± 7.2 HV1 in Base Metal A and 129.0 ± 4.2 HV1 in Base Metal B, whereas the corresponding values for GMAW/MAG were 124.5 ± 3.6 HV1 and 124.3 ± 4.7 HV1, respectively.

In both welding processes, the fusion zone (FZ) showed the highest mean hardness values. The laser-welded joints exhibited a mean FZ hardness of 158.6 ± 18.9 HV1, whereas the GMAW/MAG joints reached 190.4 ± 11.0 HV1. The increase in fusion-zone hardness relative to the base metal may be associated with the welding thermal cycle, which involves complete melting, solidification, and subsequent microstructural transformations during cooling [36].

The higher mean fusion-zone hardness observed in the examined GMAW/MAG specimens may partly reflect the contribution of the ER70S-6 filler wire. Dilution between the filler metal and the SAE 1010 base material can modify the weld-metal composition and influence its microstructural evolution and hardness [37]. Together with the different welding thermal cycles, this provides a possible explanation for the observed hardness difference.

Differences were also observed between the two sides of the heat-affected zone. For laser welding, HAZ A exhibited a mean hardness of 144.8 ± 4.3 HV1, while HAZ B reached 134.3 ± 14.2 HV1. For GMAW/MAG, the corresponding values were 136.3 ± 9.5 HV1 in HAZ A and 139.9 ± 10.3 HV1 in HAZ B. Thus, both welding processes produced local hardening within the HAZ relative to the respective base-metal regions, although the magnitude and distribution of this hardening differed between the evaluated specimens. The comparatively high standard deviations observed in some regions, particularly in the laser FZ and HAZ B, also indicate local variability along the microhardness profiles.

The regional values in Table 8 were calculated by pooling individual indentations from two independently welded specimens per process. These indentations are local measurements within specimens, not independent experimental replicates. Accordingly, the higher mean fusion-zone hardness observed in the examined GMAW/MAG specimens compared with the laser-welded specimens is a descriptive observation and does not establish a statistically significant difference between welding processes.

Values are reported as mean ± standard deviation. The parameter n represents the number of individual HV1 indentations included in each metallurgical region, pooled from two independent welded specimens for each welding process; n therefore does not represent the number of independent specimens. Regions A and B correspond to opposite sides of the welded joint. Consecutive indentations were spaced 0.5 mm apart.

Figure 7 presents the transverse microhardness profiles obtained from two independent laser-welded specimens. Both profiles showed increased hardness within the fusion zone relative to the base-metal regions. However, the magnitude of this increase varied between the two specimens, consistent with the dispersion observed in the pooled FZ values in Table 8. Local hardness variations were also observed within the HAZ regions.

Figure 8 presents the corresponding transverse microhardness profiles obtained from two independent GMAW/MAG-welded specimens. A clear local increase in hardness was observed within the fusion zone relative to the base metal, with the pooled FZ mean reaching 190.4 ± 11.0 HV1. The HAZ values remained intermediate between those of the base metal and fusion zone. Although the GMAW/MAG joints exhibited a higher mean fusion-zone hardness than the laser-welded joints, microhardness measurements alone are not sufficient to establish the presence or absence of material embrittlement. Such an assessment would require complementary mechanical and microstructural characterization.

#### Visual and Macrographic Weld Assessment

Visual and macrographic examination of representative transverse cross-sections was performed to assess weld continuity and observable discontinuities in both welding conditions. The analyzed cross-sections showed no surface cracks, visible lack of fusion, undercut, overlap, visible porosity, or non-metallic inclusions. Table 9 summarizes the results of the visual and macrographic assessment.

The main differences between the analyzed cross-sections were associated with weld geometry. The analyzed GMAW/MAG cross-section exhibited a weld-face convexity of 3.93 mm and a weld-root reinforcement of 1.51 mm. In contrast, the laser-welded cross-section exhibited a weld-face convexity of 0.56 mm, while its root reinforcement was too small to be measured reliably. The recorded penetration depths were 3.84 mm for laser welding and 4.16 mm for GMAW/MAG; these values alone were not considered evidence of full penetration. The selected laser-welded section showed a smoother weld profile, consistent with the reduced post-weld finishing requirements observed under the investigated manufacturing conditions.

These observations are restricted to the evaluated surfaces and representative transverse cross-sections. Visual and macrographic examination does not constitute a complete volumetric inspection of the entire weld length. Therefore, the absence of internal discontinuities cannot be generalized beyond the analyzed sections without complementary non-destructive testing.

### 3.6. Guided-Bend Testing

Guided-bend tests were performed to assess the continuity and deformation capability of the welded joints according to the acceptance criteria of ASME Section IX [29]. Three face-bend and three root-bend specimens were tested for each welding process (Table 10).

All specimens completed the 180° bending procedure without open discontinuities exceeding the acceptance limit of 3 mm. No visible surface cracks, tearing, or fracture were observed on the convex surfaces of the face-bend and root-bend specimens. Therefore, both the laser-welded and GMAW/MAG joints satisfied the adopted guided-bend acceptance criteria under the investigated conditions.

These results indicate that the weld metal, heat-affected zone, and adjacent base material maintained sufficient continuity to accommodate the deformation imposed during the test. However, the guided-bend test is a qualification procedure based primarily on the visual assessment of open discontinuities on the convex surface after bending and should not be interpreted as a direct measurement of tensile strength, fracture toughness, or fatigue performance [29].

**Figure 9:**
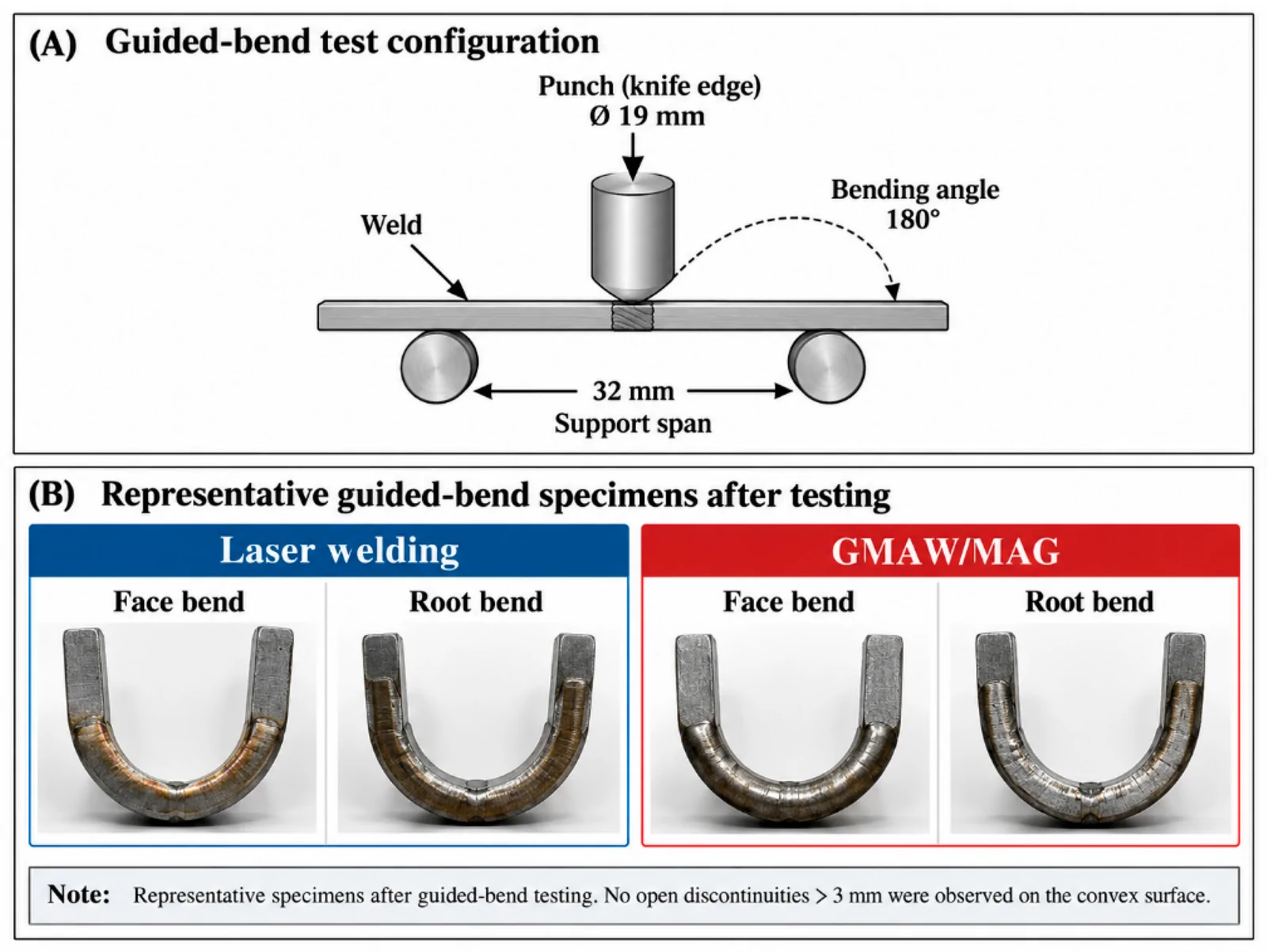
Guided-bend testing: (**A**) schematic test configuration, showing the 19 mm former diameter, 32 mm support spacing, and 180° bending angle; and (**B**) representative face-bend and root-bend specimens for laser welding and GMAW/MAG after testing.

Acceptance criterion: no open discontinuity greater than 3 mm on the convex surface of the bent specimen (Figure 9), according to ASME Section IX.

### 3.7. Industrial Forming Validation

Industrial forming validation evaluated the behavior of the welded joints under the actual manufacturing sequence used for agricultural wheel rims. Ten specimens produced by each welding process were sequentially subjected to roll bending, cone opening, and profiling. After each forming stage, the welded region was visually inspected for cracks, joint opening, fracture, geometric instability, and other discontinuities that could prevent continuation of the manufacturing process.

All laser-welded and GMAW/MAG specimens completed the initial roll-bending stage successfully. During the subsequent cone-opening operation, all ten laser-welded specimens remained intact, whereas two of the ten GMAW/MAG specimens fractured and were rejected. The eight remaining GMAW/MAG specimens proceeded to the profiling stage and completed it without additional visible failures. Consequently, the final pass rate was 100% (10/10) for laser welding and 80% (8/10) for GMAW/MAG. Table 11 summarizes the results.

**Figure 10:**
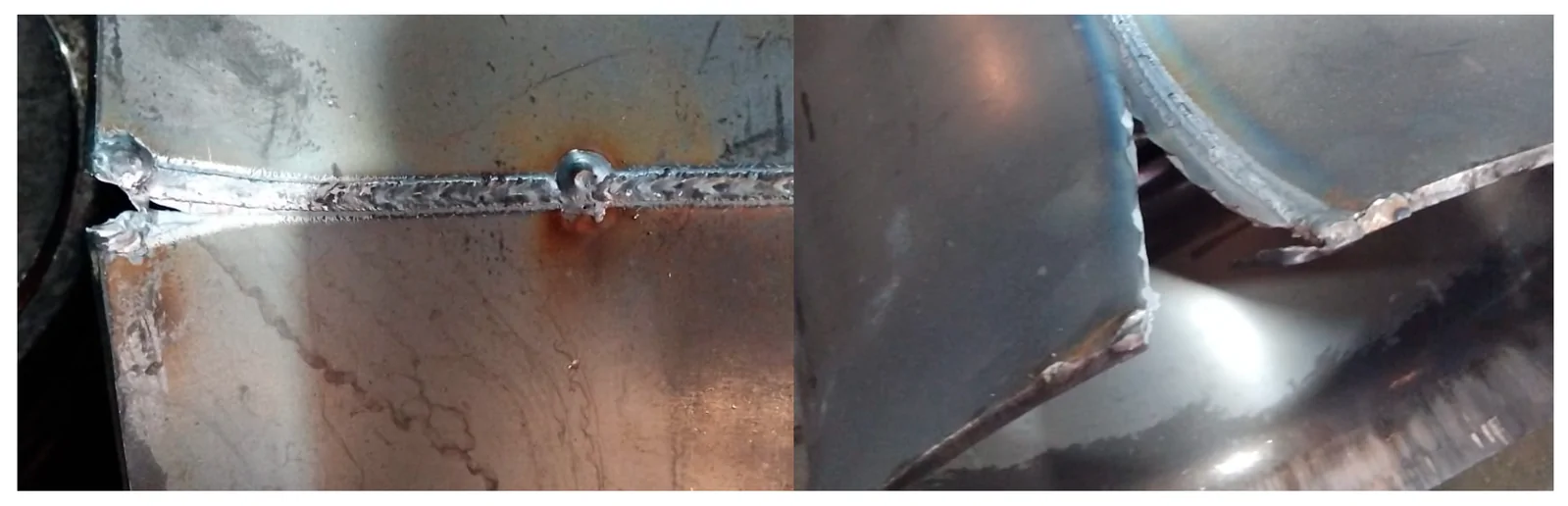
Representative GMAW/MAG-welded specimen rejected during industrial forming validation due to fracture during the cone-opening stage.

Figure 10 shows a representative GMAW/MAG specimen rejected during cone opening. The occurrence of fracture only during the second forming stage indicates that the joints withstood the initial roll-bending operation but that two GMAW/MAG specimens failed to accommodate the deformation imposed during cone opening.

The different responses observed between the welding processes may be associated with the combined effects of weld geometry and the local metallurgical conditions produced by each process. The GMAW/MAG joints exhibited substantially greater weld convexity and reinforcement, as well as higher mean hardness in the fusion zone, than the laser-welded joints. In addition, the higher energy input associated with GMAW/MAG produced a broader heat-affected zone. These characteristics may have promoted a less uniform distribution of strain during the successive forming operations, increasing local deformation near the welded region [6,19,20].

However, because no fractographic analysis, local strain measurement, tensile testing, or fracture-toughness characterization was performed on the failed specimens, a direct causal relationship between these characteristics and fracture cannot be established. Therefore, the observed failures should be interpreted as a difference in forming response under the industrial conditions investigated rather than as definitive evidence of a specific fracture mechanism.

**Figure 11:**
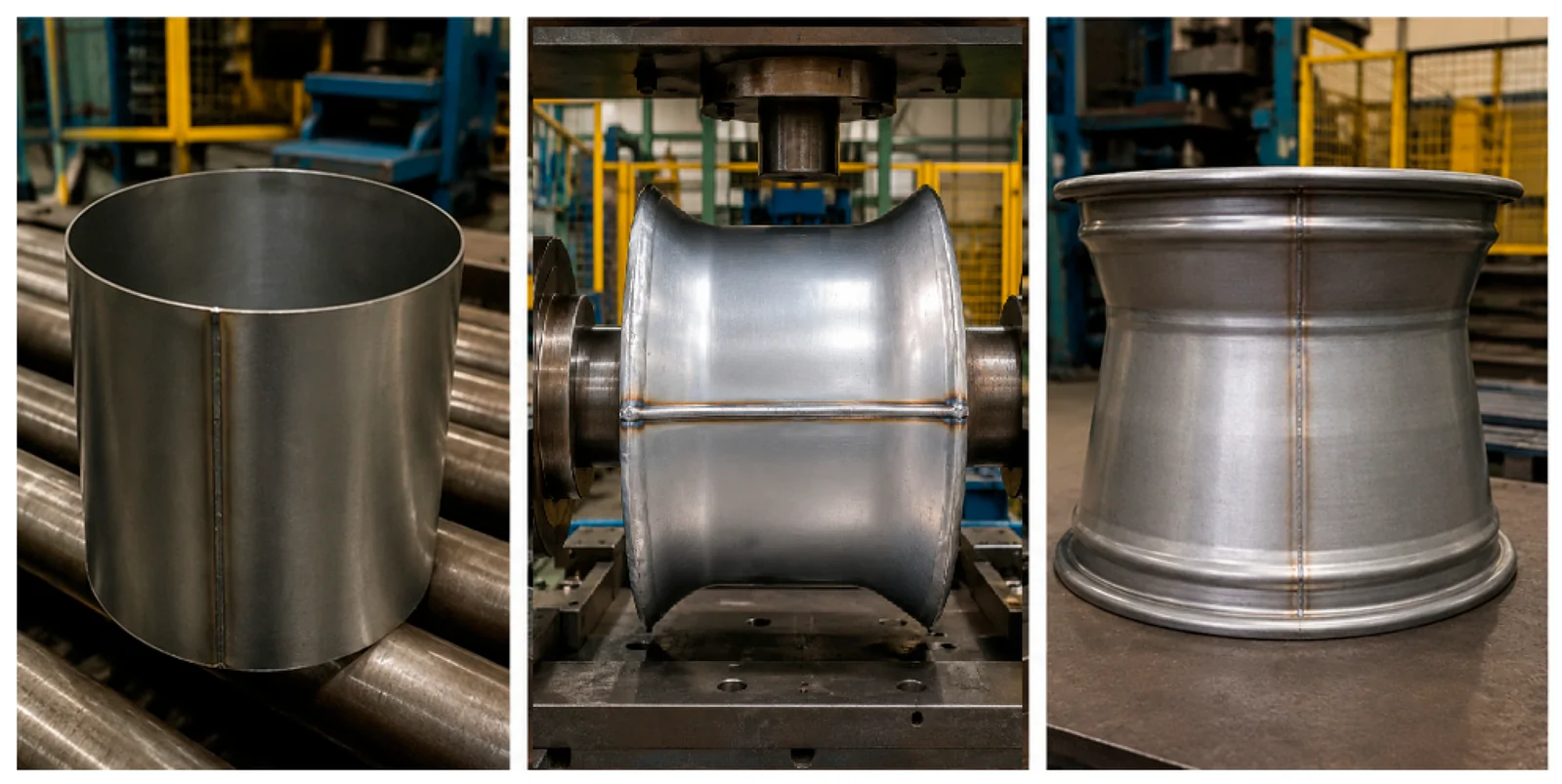
Industrial forming validation sequence: (**a**) welded component after roll bending, (**b**) cone-opening stage, and (**c**) final profiled rim.

The complete industrial forming sequence is illustrated in Figure 11. This validation complements the guided-bend tests. Although both welding processes met the guided-bend acceptance criteria under controlled laboratory conditions, the industrial forming sequence was more demanding because it subjected the welded joints to successive, cumulative deformation stages representative of the actual manufacturing route. Under these conditions, all ten laser-welded specimens completed the forming sequence, whereas eight of the ten GMAW/MAG specimens completed it.

The industrial validation revealed a difference in the observed forming response that was not apparent in the guided-bend tests. The pass rates describe the evaluated specimens and do not, by themselves, establish a statistically significant difference in production reliability.

These results are limited to the SAE 1010 steel, welding parameters, joint geometry, and forming sequence investigated in this study. Additional testing would be required before extrapolating the observed forming performance to other materials, welding conditions, or manufacturing routes.

Figure 11 illustrates the complete industrial forming sequence used in this investigation.

### 3.8. Industrial and Economic Implications

The potential operational impact of replacing conventional GMAW/MAG welding with oscillating fiber laser welding was assessed using a comparative manufacturing scenario based on production data provided by the participating company. The welding and post-weld finishing times used in this assessment correspond to standard operational times adopted by the company rather than to repeated time measurements performed as part of the present study. Therefore, these values are not reported as means and no standard deviations are applicable.

The standard welding times of 3.0 min per wheel for GMAW/MAG and 1.5 min per wheel for laser welding refer to the complete operational cycle, including workpiece positioning, clamping, welding, and removal. These values were provided by the participating company as standard production times rather than obtained from repeated experimental measurements.

The production volume adopted for the operational comparison was 10,000 wheels per month, corresponding to 120,000 wheels per year. The hourly operating costs of R$ 60.00/h for GMAW/MAG and R$ 80.00/h for laser welding correspond to company-provided reference values for 2026. These rates were supplied as aggregated operating costs, without a detailed breakdown of individual cost components. For the GMAW/MAG route, the same hourly rate of R$ 60.00/h was applied to both welding and post-weld grinding. Therefore, the estimated annual operating costs represent a preliminary comparison based on the company’s standard processing times and reference operating rates.

Capital investment, equipment depreciation, maintenance, detailed energy consumption, tooling, operator training, implementation downtime, financing costs, and payback period were not included in the calculation. Potential differences in filler-metal consumption, shielding gas, finishing consumables, rework, and scrap generation were also excluded. Consequently, the comparison presented below is restricted to processing time and the company-provided hourly operating costs.

The parameters and calculated results are summarized in Table 12. The GMAW/MAG route required a total processing time of 6.0 min per wheel, including welding and grinding, whereas the laser welding route required 1.5 min per wheel. This corresponds to a reduction of 4.5 min per wheel, or 75% of the processing time considered in this operational comparison.

Based on an annual production volume of 120,000 wheels, the estimated annual processing time was 12,000 h for the GMAW/MAG route and 3000 h for the laser welding route. Applying the hourly operating costs supplied by the participating company resulted in estimated annual operating costs of R$ 720,000 for GMAW/MAG and R$ 240,000 for laser welding. Based on the company-provided processing times and hourly rates, and assuming an annual production volume of 120,000 wheels, the estimated annual operating cost savings were R$ 480,000 for the laser welding route. This preliminary estimate is specific to the evaluated production scenario.

The reduction in processing time was mainly associated with the elimination of the post-weld grinding operation considered necessary for the GMAW/MAG route. This operational difference is consistent with the weld-geometry results presented in Section 3.3, in which the laser-welded joint exhibited substantially lower convexity and no measurable reinforcement compared with the GMAW/MAG joint.

These results should therefore be interpreted as estimates of operational performance under the investigated manufacturing scenario rather than as a complete economic evaluation. A comprehensive investment analysis would require additional information regarding equipment acquisition, depreciation, maintenance, energy consumption, production availability, financing, and payback period.

### 3.9. Integrated Interpretation of the Results

Under the investigated conditions, laser welding exhibited a lower nominal incident energy per unit length than the estimated GMAW/MAG heat input (Table 13). The selected laser-welded cross-section also showed a narrower weld-face width, lower convexity, and a smaller HAZ width than the selected GMAW/MAG cross-section. These geometric measurements represent individual sections and should not be interpreted as statistical estimates of process variability.

Both welding processes satisfied the guided-bend acceptance criteria, indicating adequate weld continuity under the imposed bending conditions. However, differences became evident during industrial forming. All ten laser-welded specimens completed the manufacturing sequence, whereas two of the ten GMAW/MAG specimens fractured during cone opening. Post-weld grinding was not required for the laser-welded specimens under the evaluated production conditions.

Overall, the results indicate that the principal advantage of laser welding was not associated with a single isolated characteristic, but with the combined reduction in thermal and geometric discontinuities. These characteristics were accompanied by a more consistent response during industrial forming and reduced post-weld finishing requirements. Nevertheless, the observed relationships should be interpreted within the investigated material, welding parameters, and manufacturing conditions, and they do not establish direct causal relationships with long-term mechanical performance.

### 3.10. Study Limitations

The results of this study should be interpreted within the experimental and industrial conditions investigated. The analysis was conducted using 4.25 mm thick SAE 1010 steel, one oscillating laser-welding condition, and one industrial GMAW/MAG reference condition. Therefore, the findings should not be directly extrapolated to other materials, sheet thicknesses, joint geometries, welding parameters, or manufacturing routes without additional experimental validation.

The welded joints were evaluated through macrographic examination, optical microscopy, grain-size measurements, Vickers microhardness testing, guided-bend testing, and industrial forming validation. Although these methods provided complementary information on weld geometry, microstructural evolution, local hardness, weld continuity, and manufacturing response, they did not constitute a complete mechanical characterization. Tensile, fatigue, impact, fracture-toughness, residual-stress, and fractographic analyses were not performed. Consequently, the present results do not allow conclusions regarding long-term structural performance or the mechanisms responsible for the failures observed during cone opening.

Quantitative weld-geometry measurements were obtained from one transverse cross-section per welding condition. Therefore, the reported geometric differences describe the selected sections and do not quantify within-process variability or establish statistical significance. Additional independently welded specimens and cross-sectional measurements would be required to assess the repeatability of these geometric characteristics.

The weld-quality assessment was based primarily on visual inspection and selected transverse cross-sections. Therefore, the absence of porosity, inclusions, lack of fusion, or other internal discontinuities should be understood as limited to the analyzed regions. Future work could include complementary volumetric non-destructive testing, such as radiographic or ultrasonic inspection, to extend the assessment beyond the examined sections.

The operational assessment was also limited to the manufacturing scenario provided by the participating company. The analysis considered welding and post-weld finishing times, representative production volume, and hourly operating costs, but did not include capital investment, equipment depreciation, maintenance, energy consumption, tooling, operator training, implementation downtime, or payback period. Accordingly, the estimated savings should be interpreted as a comparative operational scenario rather than as a complete techno-economic evaluation.

Despite these limitations, the combination of metallurgical characterization, standardized qualification testing, industrial forming validation, and operational assessment provides a consistent basis for comparing the investigated welding processes under the evaluated conditions. Further studies including additional welding parameters, mechanical testing, non-destructive inspection, and long-term service evaluation would be required to extend the applicability of the present findings.

In addition, the local sheet thickness, penetration-measurement endpoints, and original image calibration require verification before the recorded penetration depths can be used to establish full penetration.

## 4. Conclusions

This study demonstrated the technical feasibility of oscillating fiber laser welding as an alternative to conventional GMAW/MAG welding for 4.25 mm thick SAE 1010 agricultural wheel rims under the investigated manufacturing conditions.

Laser welding exhibited a nominal incident energy per unit length of 0.019 kJ/mm, compared with an estimated heat input of 0.64 kJ/mm for GMAW/MAG. In the analyzed transverse cross-sections, the laser-welded joint showed a smaller weld-face width (5.70 versus 12.97 mm), lower convexity (0.56 versus 3.93 mm), and a narrower HAZ (0.85 versus 2.10 mm). These geometric observations are descriptive because one cross-section was quantitatively evaluated per welding condition. The recorded penetration depths of 3.84 mm for laser welding and 4.16 mm for GMAW/MAG, relative to a nominal sheet thickness of 4.25 mm, were not sufficient on their own to confirm full penetration. Under the evaluated manufacturing conditions, the laser-welding route also eliminated the post-weld grinding operation required for the conventional GMAW/MAG route.

The microstructural and microhardness results revealed differences consistent with the thermal cycles associated with the two processes. The highest mean hardness occurred in the fusion zone, reaching 158.6 ± 18.9 HV1 for laser welding and 190.4 ± 11.0 HV1 for GMAW/MAG. Both welding conditions passed the guided-bend tests, with 3/3 face-bend and 3/3 root-bend specimens satisfying the adopted acceptance criterion for each process. These results demonstrate compliance with the adopted bend-test acceptance criterion, rather than providing direct measurements of tensile strength, fracture toughness, or fatigue performance.

Industrial forming provided a clearer distinction between the welding conditions. All laser-welded specimens completed the roll-bending, cone-opening, and profiling sequence, resulting in a final pass rate of 10/10 (100%). For GMAW/MAG, two specimens fractured during cone opening, resulting in a final pass rate of 8/10 (80%). This result highlights the importance of evaluating welded joints under the actual manufacturing sequence in addition to standardized qualification testing.

Based on standard operational times and hourly operating costs provided by the participating company, the comparative production scenario indicated a reduction in processing time from 6.0 to 1.5 min per wheel with laser welding. This operational estimate does not constitute a complete economic analysis because capital investment, depreciation, maintenance, detailed energy consumption, and payback period were not considered.

Overall, the industrial forming tests provide the principal evidence of manufacturing feasibility under the investigated conditions: all ten laser-welded specimens completed the forming sequence, compared with eight of ten GMAW/MAG specimens. The geometric measurements from one transverse section per welding condition provide descriptive observations that complement these forming results, without establishing geometric repeatability. These findings remain limited to the evaluated material, welding parameters, and manufacturing sequence.

These findings support manufacturing feasibility under the investigated conditions. Long-term structural performance, including fatigue, impact behavior, fracture toughness, and residual stresses, remains outside the scope of this study.

## Figures and Tables

**Table 1:** Measured chemical composition of SAE 1010 steel determined by optical emission spectrometry (wt.%).

Element	C	Mn	P	S	Fe
SAE 1010	0.12	0.50	0.02	0.025	Balance

**Table 2:** Welding parameters used for the GMAW/MAG and laser welding processes.

Parameter	GMAW/MAG	Laser Beam Welding (LBW)
Sheet thickness (mm)	4.25	4.25
Number of passes	1	1
Welding current (A)	285	Not applicable
Arc voltage (V)	32	Not applicable
Laser power (kW)	Not applicable	2.95
Welding speed (mm/s)	11.33	155
Filler metal	ER70S-6, Ø1.6 mm	None
Wire-feed speed (cm/min)	42	Not applicable
Shielding gas	90% Ar 10% CO_2_	N_2_ (99.999%)
Gas flow rate (L/min)	23	20
Programmed oscillation amplitude (mm)	Not applicable	5.2
Oscillation frequency (Hz)	Not applicable	2000
Oscillation pattern	Not applicable	Linear transverse
Root gap (mm)	≤0.15	≤0.15

**Table 3:** Summary of the experimental procedures and corresponding standards or reference methods.

Experimental Procedure	Standard/Reference
Macrographic examination and metallographic specimen preparation	ISO 17639:2023 [30]; ASTM E3 [31]
Microstructural characterization and grain size measurement	ASTM E407 [32]; ASTM E112 [27]
Vickers microhardness testing	ASTM E384 [28]
Visual inspection and weld-quality assessment	ISO 17637 [33]; ISO 13919-1 [34]; ISO 15614-11 [35]
Guided bend testing	ASME Section IX (QW-162, QW-163 and QW-462.3(a)) [29]
Industrial forming validation	Industrial manufacturing procedure adopted by the company

**Table 4:** Decision matrix used for welding-process selection.

Essential Criterion	Weight	Current GMAW/MAG	Weighted Score	Optimized GMAW/MAG	Weighted Score	Laser Welding	Weighted Score
Reduced HAZ	5	1	5	2	10	5	25
Penetration control	5	2	10	3	15	5	25
Integrity after forming	5	1	5	3	15	5	25
Repeatability	4	2	8	3	12	5	20
Hardness variation	4	1	4	3	12	5	20
Ease of industrial integration	3	2	6	3	9	5	15
Total weighted score			38		73		130

Note: Criterion weights and alternative ratings were assigned by the author based on the technical requirements of the application. Ratings range from 1 (very poor) to 5 (very good), with higher values indicating more favorable expected performance. Optimized GMAW/MAG represents a conceptual process-improvement alternative that was not experimentally validated. The weighted scores reflect an engineering assessment used for technology selection, rather than measured experimental results.

**Table 5:** Estimated energy input per unit length for the evaluated welding processes.

Parameter	GMAW/MAG	Laser
Nominal power input (kW)	9.12	2.95
Welding speed (mm/s)	11.33	155
Assumed thermal efficiency	0.80	Not applied
Estimated energy input per unit length (kJ/mm)	0.64	0.019

**Table 6:** Geometric measurements obtained from one transverse cross-section per welding condition (n = 1).

Parameter	Laser Welding	GMAW/MAG
Nominal sheet thickness (mm)	4.25	4.25
Penetration (mm)	3.84	4.16
Weld-face width (mm)	5.70	12.97
Weld-face convexity (mm)	0.56	3.93
Weld-root reinforcement (mm)	Not measurable	1.51
HAZ width (mm)	0.85	2.10
Visible surface discontinuities	None	None

Note: Weld-face convexity and weld-root reinforcement refer to geometric features on opposite sides of the welded joint. “Not measurable” indicates that the root reinforcement in the analyzed laser-welded cross-section was too small to be quantified reliably using the adopted measurement procedure; it does not imply a value of zero.

**Table 7:** Grain-size measurements according to ASTM E112 [27].

Region	ASTM Grain Number	Mean Intercept Length (µm)
Laser welding, BM	10.64 ± 0.10	8.02 ± 0.30
Laser welding, HAZ	10.80 ± 0.12	7.59 ± 0.35
GMAW/MAG, BM	10.55 ± 0.11	8.25 ± 0.40
GMAW/MAG, HAZ	8.80 ± 0.25	16.40 ± 1.80

**Table 8:** Vickers microhardness values measured in the different metallurgical regions of the laser-welded and GMAW/MAG joints.

Region	Laser (HV1)	n	GMAW/MAG (HV1)	n
Base Metal A	131.9 ± 7.2	10	124.5 ± 3.6	8
HAZ A	144.8 ± 4.3	4	136.3 ± 9.5	12
Fusion Zone (FZ)	158.6 ± 18.9	24	190.4 ± 11.0	18
HAZ B	134.3 ± 14.2	4	139.9 ± 10.3	16
Base Metal B	129.0 ± 4.2	10	124.3 ± 4.7	11

**Table 9:** Results of the visual and macrographic assessment of the evaluated welded joints.

Evaluated Characteristic	Laser Welding	GMAW/MAG	Inspection Method
Surface cracks	Not observed	Not observed	Visual and macrographic examination
Visible lack of fusion	Not observed	Not observed	Macrographic examination
Non-metallic inclusions	Not observed	Not observed	Macrographic examination
Visible porosity	Not observed	Not observed	Macrographic examination
Undercut	Not observed	Not observed	Visual and macrographic examination
Overlap	Not observed	Not observed	Macrographic examination
Weld-face convexity (mm)	0.56	3.93	Macrographic measurement
Weld-root reinforcement (mm)	Not measurable	1.51	Macrographic measurement
Measured penetration (mm)	3.84	4.16	Macrographic measurement
Overall observation	No visible discontinuities of the evaluated types in the analyzed cross-section	No visible discontinuities of the evaluated types in the analyzed cross-section	Visual and macrographic examination

**Table 10:** Results of guided-bend tests performed according to ASME Section IX.

Welding Process	Bend Condition	Specimens Tested	Open Discontinuities > 3 mm	Maximum Observed Discontinuity	Specimens That Passed	Result
Laser welding	Face bend	3	None	None observed	3/3	Pass
Laser welding	Root bend	3	None	None observed	3/3	Pass
GMAW/MAG	Face bend	3	None	None observed	3/3	Pass
GMAW/MAG	Root bend	3	None	None observed	3/3	Pass

**Table 11:** Results of the industrial forming validation.

Manufacturing Stage	Laser Welding	GMAW/MAG	Acceptance Criterion
Roll bending	10/10 passed	10/10 passed	No visible cracks, fracture, or joint opening
Cone opening	10/10 passed	8/10 passed	No visible cracks, fracture, joint opening, or geometric instability
Profiling	10/10 passed	8/8 passed *	No visible cracks, fracture, joint opening, or dimensional instability
Final result	10/10 passed (100%)	8/10 passed (80%)	Successful completion of the complete manufacturing sequence

* Only the eight GMAW/MAG specimens that passed the cone-opening stage proceeded to profiling.

**Table 12:** Estimated operational comparison between GMAW/MAG and laser welding under the evaluated manufacturing scenario.

Parameter	GMAW/MAG	Laser Welding
Standard welding time per wheel (min)	3.0	1.5
Standard grinding time per wheel (min)	3.0	0
Total processing time per wheel (min)	6.0	1.5
Monthly production volume (wheels)	10,000	10,000
Annual production volume (wheels)	120,000	120,000
Estimated annual processing time (h)	12,000	3000
Company-provided hourly operating cost (R$/h)	60.00	80.00
Estimated annual operating cost (R$)	720,000	240,000
Estimated annual operational cost saving (R$)	Not applicable	480,000

Note: Welding times represent complete operational cycles, including workpiece positioning, clamping, welding, and removal. Welding and grinding times are standard production values provided by the participating company and were not obtained from repeated experimental measurements; therefore, standard deviations are not applicable. Hourly operating costs correspond to aggregated company-provided reference rates for 2026, without a detailed breakdown of cost components. The GMAW/MAG hourly rate of R$ 60.00/h was applied to both welding and grinding, whereas the laser welding rate was R$ 80.00/h. Annual estimates were calculated assuming a production volume of 120,000 wheels.

**Table 13:** Comparative summary of the evaluated welding processes.

Parameter	Laser Welding	GMAW/MAG	Main Observation
Estimated energy input per unit length (kJ/mm)	0.019	0.64	Lower nominal incident energy per unit length for laser welding
Weld-face width (mm) in the examined laser-welded cross-section	5.70	12.97	Narrower weld profile for laser welding
Weld convexity (mm) in the examined laser-welded cross-section	0.56	3.93	Lower geometric discontinuity for laser welding
HAZ width (mm) in the examined laser-welded cross-section	0.85	2.10	More restricted HAZ for laser welding
HAZ mean intercept length (µm)	7.59 ± 0.35	16.40 ± 1.80	Less grain coarsening in the laser-welded HAZ
Guided-bend testing	All specimens passed	All specimens passed	Both processes satisfied the adopted acceptance criteria
Industrial forming validation	10/10 passed	8/10 passed	More consistent forming response for laser welding
Post-weld grinding	Not required	Required	Lower finishing requirement for laser welding

Note: Weld-face width, weld convexity, and HAZ width correspond to measurements from one transverse cross-section per welding condition (n = 1). These values are descriptive and do not represent means across independently welded joints.

## Data Availability

The original contributions presented in this study are included in the article. Further inquiries can be directed to the corresponding author.
